# Career Orientations of Pre-Service Teachers: Exploring the Influence of Different Types of Universities

**DOI:** 10.11621/pir.2024.0208

**Published:** 2024-06-30

**Authors:** Roza A. Valeeva, Gulfiia G. Parfilova, Faina M. Kremen, Sergei A. Kremen

**Affiliations:** a *Kazan Federal University, Russia*; b *Federal Scientific Center of Psychological and Multidisciplinary Research, Kazan Branch, Russia*; c *Smolensk State University, Russia*

**Keywords:** teaching profession, teacher education, professional career, career orientations, student of pedagogical bachelor degree, federal university, regional university

## Abstract

**Background.:**

More than half of future teachers in our country receive professional training in classical universities, which determines a great variability of motivational-professional and planned career trajectories. At the same time, the type of university (federal or regional) significantly influences the conditions for forming a professional teacher. This study identifies career orientations of students attending classical universities of different types in order to determine trends in career preferences according to choice of university type.

**Objective.:**

This study aims to identify the peculiarities of career orientations of students studying at pedagogical bachelor degree programs of classical universities of different types (federal and regional), determining the interrelations of their indicators with socio-demographic and motivational-professional characteristics of the test subjects.

**Design.:**

The study was conducted in the form of a comparison of career orientations of the first-year students in teacher education programs (83 students of Kazan Federal University and 89 students of Smolensk State University). The empirical methods used included the adapted Schein’s career anchors tool and a questionnaire revealing socio-demographic and motivational-professional variables to identify factors related to career orientations.

**Results.:**

The obtained results revealed common preferences for both universities in career orientations on the *service*, job stability and *lifestyle integration* scales, indicating stable trends in choosing the teaching profession. Students at both universities, who chose the teaching profession and plan to work in their specialty, demonstrate a readiness for professional development and overcoming difficulties. However, the university is an independent factor which determines differences according to the scales of *entrepreneurship*, *autonomy* and *management*. Students at a federal university are characterized by greater independence and initiative, but also more uncertainty in choosing a profession, in contrast to students at a regional university.

**Conclusion.:**

Considering student career orientations according to their socio-demographic and motivational-professional characteristics, allows us to obtain an objective and comprehensive picture of the professional choices of students from different types of universities, leading to more effective delivery of their professional development.

## Introduction

By the beginning of the 21st century, most of the developed world has established diverse and flexible systems of teacher education. Meanwhile, the problems of teacher shortage and retention are still relevant (Watt & Richardson, 2023). In Russia, there is a shortage of teachers in schools despite a sufficient number of graduates in pedagogical specialties. A significant number of young teachers change their field of activity in the first years of work. These issues highlight the need for conscious and purposeful self-determination during professional education, since professional choice and professional education are closely interrelated and interdependent. Future career opportunities are determined by both the choice of training direction and the institution where the professional qualification is obtained.

In 2023, teacher training programs lasting four or five years were offered in 285 Russian universities (Vuzoteka, 2023). However, more than half of the country’s students receive education not in specialized pedagogical universities but in traditional ones (Nazarov & Soboleva, 2020), which, due to the optimization processes of higher education, are categorized into federal, research, and regional universities. The Federal State Educational Standard for higher education ensures substantive consistence for teacher preparation programs across the university sector. However the type of university has an effect on factors such as faculty qualifications, opportunities for using innovations, and the material and technical equipment of the educational process, which collectively influences the quality of education (Shibanova et al., 2023). The diversity of pedagogical programs creates conditions for obtaining professional training in universities with different levels and statuses.

This study questions the impact of attending different types of universities for pedagogical education on students’ career orientations, including their value perceptions of the profession, development prospects and motivation to work as a teacher (Valeeva et al., 2022).

This study is based on the *anchor model of professional development* (Super, 1990; Holland, 1997; Schein, 1990), within which career orientations are an element of self-concept, reflecting the direction of professional advancement chosen by the individual, based on their needs, motives, interests, abilities, and the social-cognitive career theory (Lent et al., 1994), which focuses on the interaction between people, their behavior, and the environment. According to this theory, the professional development of an individual is formatively influenced by the environment in which he realizes his agency (Minina et al., 2021).

### Career orientations. Schein’s Career Anchor Theory

In recent decades, the focus in career research has increasingly shifted towards studying the internal or subjective characteristics of a person: values, attitudes, and beliefs, rather than organizational aspects (Cappellen & Janssens, 2010). Highlighting these subjective elements in the career structure has focused attention on the study of career orientations, which, in the context of professional self-determination and professionalization, are considered a semantic disposition that serves to prioritize the direction of professional advancement and holds stable life meaning for the individual (Zhdanovich, 2008).

Schein proposed the concept of a *career anchor*, that is, “a combination of perceived areas of competence, motives, and values that [an individual] would not give up [because] they represent [his or her] real self” (Schein, 1990, p. 1). Career anchors are stable and long-term factors and reflect an individual’s understanding of their strengths, weaknesses, competencies, value system and vision of a desired career. They are formed through accumulated experiences involving learning and self-development processes, family and environmental influences. The fit between career anchors and the work environment leads to positive outcomes such as job performance and job satisfaction (Schein, 1996).

The conceptualization of Schein’s ideas involved examining how different anchors combine their complementarity, or incompatibility. Ramakrishna & Potosky (2003) showed in their study that proximity or opposite anchors have different effects on career outcomes. Wils et al. (2014) systematized the classification by identifying two perpendicular axes that form four quadrants: bureaucratic, change, careerist, and social. The first axis denotes the poles of individual and collective orientation, and the second axis denotes normative and affective orientation. Accordingly, the orientations included in the social quadrant are based on collective values and values of social relations. Researchers tested this model on employees working in different fields including, healthcare (Wils et al., 2014), management (Wils et al., 2016), and IT professionals (Igbaria & Baroudi, 2012; Chang & Wu, 2022). There has been no research on teacher career orientation in the context of this framework of analysis, although it can be assumed that career anchors, specific to the teaching profession, are concentrated in the social quadrant.

Russian researchers (Egorova, 2017; Sheveleva, 2019; Solovyova & Zausenko, 2015; Tsaritsentseva, 2014; Yurtaeva, 2012) used an adapted Schein’s questionnaire for identifying career orientations among universities students. Career orientation in students is characterized by insufficient awareness and depend on the orientation and period of professional training (Polyanskaya & Ernazarova, 2019). However, Bukova & Chiker (2022) confirmed the relationship between career orientations and orientation but did not find statistically significant differences depending on the course of study.

### Factors influencing career choices

Researchers have identified individual and contextual factors that influence career choices. Super (1990) argues that both individual (professional values, abilities, needs), and environmental factors (the influence of significant others) influence career choices. Socio-cognitive career theory (Lent et al., 1994) as a strand of Bandura’s (1986) general socio-cognitive theory substantiate the relationship of vocational interest formation and academic and career choices and performance in educational and professional endeavors. They identify personal and extra-personal factors that help individuals develop their careers. The former include self-efficacy, expected outcomes, and goal-setting mechanisms, which may be influenced by gender, support from loved ones, learning experiences, and other contextual factors (Lent et al., 1994).

### Personal factors

Research has extensively explored the relationship between personality characteristics and different aspects of career direction (Lent et al., 2019), identifying which personality traits (Lounsbury et al., 2005) and personal values (Fearon et al., 2018) are prerequisites for university students’ career choices. Coleman et al. (2023) identified personality traits that influence career decisions and career indecision. Lounsbury et al. (2005) revealed that career choice is positively correlated to subjective well-being and successful transition from school to work. Students who are more deliberate in successfully pursuing career opportunities in their chosen career field tend to have higher levels of work-life satisfaction (Li et al., 2019).

Motivation has a significant influence on the formation of professional identity, beliefs, and opportunities in teacher career development (Thomson & Palermo, 2014). Traditionally, altruistic, intrinsic, and extrinsic motivations are considered the primary ones for the teaching profession. Altruistic motives such as serving society, contributing to its development and helping and supporting students are the most important factor in choosing the teaching profession. Internal motives are also significant and include an inherent interest in teaching, pleasure from the activity, an interest in the subject area of instruction, desire to work with children, and opportunities for professional or personal self-development. External motives include opportunities to combine work and family, job security and good working conditions (Fray & Gore, 2018).

Watt and Richardson (2007) developed the *factors influencing teaching choice* methodological approach (FIT-Choice). Numerous studies have identified that the most significant of these factors include, the values of social utility and intrinsic value of teaching, self-assessment of teaching abilities, positive previous experience of teaching and learning, values of personal utility. Teaching as a fallback and social influence is found to be the least effective (Fray & Gore, 2018).

A number of studies have examined the relationship of extrinsic and intrinsic values with the career management processes of university students. Jackson and Tomlinson (2019) found the leading importance of intrinsic career values in career planning. Sortheix et al. (2013) showed the relationship of intrinsic career values with work engagement.

The explanation of career decision making based on Holland’s interest typology (Holland, 1997) involves finding a match between one’s skills and interests and the profile of the relevant specialty (Nauta, 2010; Nye, et al., 2017). Students tend to enter higher education based on subject interest without having decided on a career path (Vulperhorst et al., 2020). Research by Quinlan et al. (2022) shows that interest in a subject shapes the desire to maintain and develop it in a future career, ensuring pro-activity in learning activities.

### Contextual factors

Contextual factors also have a significant impact on career choice and development. In particular, culture (Guan et al., 2018), gender and past experiences (Lee et al., 2023) are significant. Other studies have linked social status (Duffy et al., 2018), social support (Dalla Rosa et al., 2019) and family influence (Marks et al., 2018) to career vocation. The influence of these contextual factors on young people’s career self-efficacy, outcome expectations, career interests and goals has been proven (Kenny & Medvide, 2013).

Contextual factors influencing teacher career preference have sociocultural differentiation. In particular, in traditional cultures, a career as an educator is more in line with the female role because it is easier to combine it with family roles (Fray & Gore, 2018).

There are practically no studies that consider the choice of university for obtaining a profession as an independent variable. Most research has focused on the career expectations and intentions of foreign students (Ayoobzadeh et al., 2021). Ishakove and Kosheleva (2023) construct a typology of students’ career trajectories in the correlation of local and global aspects. Bukova and Chiker (2022) compare career orientations of Russian students studying in various disciplines in metropolitan and regional universities. Al Tamimi et al. (2023) found that when choosing a university its reputation is a more significant factor compared to location and infrastructure. Examples of research on students’ career orientations, considering the specific characteristics of the university (specialized, classical, federal, regional) are sporadic (Valeeva et al., 2022).

### The combination of personal and contextual factors in choosing a teaching program and a university

It is obvious that there is a close relationship between the choice of specialty and the choice of university, but it is reasonable to consider them separately, as they are conditioned by different factors.

According to Russian studies, the choice of direction for applicants is on average more important than the choice of university (Shibanova et al., 2023). Teacher education remains one of the most popular, geographically widespread and provided with budgetary places in the Russian structure of higher education. Preservation of the average score (68 points) (Quality of admission to Russian universities, 2022) along with an increase in the number of applicants for both budget and extra-budget places testifies to the unchanged demand for teacher education in the regional labor markets, and the presence of its *target group* of applicants (Quality of admission to Russian universities, 2021). Despite the positive dynamics of the passing score, the pedagogical field remains less prestigious and is in demand primarily among families with less cultural capital (Shibanova et al., 2023). The influence of socioeconomic status, education, and professional status of parents on career and educational choices is proven in a number of foreign (Ginevra et al., 2015) and Russian (Khavenson & Chirkina, 2019) studies.

However, individual factors such as an interest in a particular field which matches one’s aptitudes, abilities, and school performance often mediate the influence of family socioeconomic characteristics (Bogdanov & Malik, 2020).

In Kuzmina’s study (2013), students from less educated and well-offfamilies were more prevalent among those in pedagogical specialties compared to engineering and economic specialties. At the same time, the students’ motives for entering the pedagogical specialty include the needs of personal development, gaining new knowledge, the importance of the future profession, and external motives such as ease of entry or training and the desire to increase their social status (Kuzmina, 2013; Shibanova et al., 2023). The last group of motives, where the choice of university and specialty is primarily driven by the guarantee of obtaining a higher education diploma, is not uncommon for Russian entrants. However, this decision is often ineffective and limits their further career opportunities (Minina & Pavlenko, 2023). Another specific feature characteristic of teacher education is the perception of the teaching profession as predominantly female, which obviously affects the choice of this field by young people of different genders (Kremen & Kremen, 2021).

When choosing a university, a significant factor is the reputation of the educational organization, traditionally associated with the quality of education. This quality encompasses a wide range of parameters: the level of qualification of the faculty, technological equipment of the educational process, employment prospects of graduates (Shibanova et al., 2023).

If we talk about teacher training in classical universities, it is obvious that universities of different types: federal and regional have unequal opportunities to implement teacher education programs. The task of federal universities is to carry out scientific and innovative activities. Accordingly, the training of teachers in such universities is carried out with a wide use of the most modern educational technologies, integration of science and practice on the basis of experimental sites, which are the best educational institutions in the regions. Located in large cities, these universities are attractive for young people not only in their region and federal district, but also in the whole country. However, the most common type of classical universities is regional universities where teacher education has an *intra-regional* character and is characterized by a low level of education migration between regions (Nazarov & Soboleva, 2020).

Indicators of high quality activities in higher education institutions (HEI) include their ranking their special status, inclusion in various state projects, the Unified State Exam (USE) pass rates, competition for admission, and demand for graduates in the labor market. Higher status and prestigious HEIs are perceived as more selective. In Russian universities, selectivity is characterized by the absence of specific requirements imposed on applicants for admission (Bugakova & Prakhov, 2021). The key indicator of selectivity is an average USE score of at least 70 points, which indicates the quality of professional training through the demand for educational programs by applicants with high academic performance (Malinovskii & Shibanova, 2023).

Not only academic performance and USE results, but also various family characteristics influence the choice of a university. Families with higher incomes and educational and professional status are willing to financially support their children if budget places are unavailable (Shibanova et al., 2023) and/or moving to another city is required. Families with medium and high socioeconomic status are more interested in educating their children at prestigious, selective universities (Eldegwy et al., 2022; Khavenson & Chirkina, 2019). Conversely, a low financial status acts as an external barrier to enroll in paid education or move to another region. Given that selective universities are not available in every region, the territorial factor can become a serious barrier to choosing a university (Malinovskii & Shibanova, 2023), not only because of financial difficulties, but also because of problems with psychological adaptation to another city. It is obvious that applicants with low USE scores from families with medium and low economic prosperity prefer programs with budgetary places at universities in their regions. According to the longitudinal study *Trajectories in Education and Profession* (Shibanova et al., 2023), applicants with an average USE score below 70 more frequently cite not only the quality of education but also their interest in a particular profession. This motivation may be conditioned by the certainty of career trajectory, which guarantees a job after graduation.

### Purpose of the study

To identify the peculiarities of career orientations of students studying at pedagogical undergraduate programs of classical universities of different types (federal and regional), and to determine the interrelations of their indicators with socio-demographic and motivational-professional characteristics of the test subjects.

### Research Questions

1. What are the differences in socio-demographic and motivational-professional characteristics of students enrolled in pedagogical programs in federal and regional universities?
2. Is there a single “career profile” specific for student-future teachers, regardless of the type of university?
3. Are social-demographic and motivational-professional characteristics linked to career orientations of students studying at universities of different types?
4. Is the type of university an independent factor determining differences in students’ career orientations?

## Methods

### Characteristics of the study institutions

Kazan (Volga Region) Federal University (KFU) is the central university in the Volga Federal District, the largest federal university with more than forty eight thousand students from Russia and abroad. According to the international QS and World University Rankings, the University is among the top three Russian universities in Education. More than nine thousand students are are enrolled in *Education and Pedagogical Sciences*. The University offers different models of teacher education. Traditional model is developed by strengthening the personnel, laboratory and information base. The distributed model is based on combining the capabilities of a classical university (fundamental training) and a pedagogical university (psychological, pedagogical and methodological training). Within the framework of the integrative model, variable educational trajectories are created for students and graduates entering the teaching profession from non-pedagogical training programs.

Smolensk State University (SmolSU) is the largest higher education institution in the region, offering a wide range of educational programs designed primarily for students from its own and neighboring regions. About five thousand students study at the university, 52% of them study on thirty five bachelor and master degree teacher training programs, implemented in eight faculties. The traditional model of teacher training prevails at the university, while elements of the integrative model are being developed.

In both universities offer budgetary and extra-budgetary places for admission to teacher education programs (bachelor’s degree), with the same list of USE disciplines and minimum scores required for admission. The results of enrollment (see *Table 1*) suggest that teacher education at KFU is selective; the choice of this university among applicants with high scores indirectly indicates its high quality and established reputation t the national level. The average score for pedagogical programs at SmolSU slightly exceeds the average score for the country, indicating the university’s stable position at the regional level.

**Table 1:** Enrollment in teacher education programs in 2022*

	Budget people admission, people	Average score	Paid admission, people	Average score
KFU	404	80.5	278	71.6
SmolSU	198	69.8	50	64.3

* According to the website “Monitoring the Quality of Admission to Higher Education Institutions” (https://ege.hse.ru/rating/2022/91645072/all/)

### Participants

The study was conducted among first-year undergraduate students at Kazan Federal University and Smolensk State University, majoring in *44.03.01 Teacher Training* and *44.03.05 Teacher Training* with two training profiles. At Kazan Federal University, students from all pedagogical programs of the Institute of Psychology and Education participated in the study: eighty three students from the programs, *Preschool Education*, *Primary Education and Foreign (English) Language*, and *Additional Education and Foreign (English) Language*. At Smolensk State University the study was conducted within the Faculty of Philology, where a total of eighty nine students from the programs, *Russian Language and Literature* and two foreign languages were surveyed. The number of girls: seventy five (90.4%) in KFU and eighty four (94.4%) in SmolSU. Average age of students: 18.6 years (SD - 0.695) in KFU and 18.5 years (SD — 0.692) in SmolSU.

All students were informed about the purpose of the study and participated on a voluntary basis. Anonymity and confidentiality were guaranteed to the participants.

### Research Methods

To study students’ career orientations, we chose the *Career Anchors* questionnaire by E. Schein (Chiker & Vinokurova, 2006), which is used to determine the main professional motives, value orientations, and social attitudes towards career and work. The methodology measures, on a 10-point scale, the level of eight career orientations: professional competence, management, autonomy, stability (place of work and place of residence), service, challenge, lifestyle integration of and entrepreneurship.

To determine the influence of various factors (socio-demographic and motivational-professional), a questionnaire was made, including the following variables: gender, form of education, economic status of the family, region of residence, place of residence, reasons for enrollment in this program, experience of pedagogical activity, professional plans.

### Procedure

The collection of data was utilized the *Google Forms* service. The link to the survey was distributed to those first year students selected for the study. The instructions for the test indicated that participants should evaluate the statements in the context of the teaching profession. The study was conducted in May, 2023 at the end of the second academic semester.

### Analysis

When analyzing the data, the sample was divided by universities according to the research design. The collected data were analyzed using IBM SPSS Statistics 23. First, the distribution of values of independent variables (construction of frequency distribution) was determined for comparison between the two groups. The method of hierarchical log-linear analysis was used to determine the relationships between nominal variables. Next, the mean values of nine career orientations were compared using the t-test for independent samples: (1) to compare the samples of two universities as a whole; (2) to determine significant differences for subgroups distinguished by socio-demographic and motivational-professional characteristics. To identify interrelationships in the structure of career orientations when comparing two samples, it was decided to abandon correlation analysis in favor of factor analysis (with varimax rotation) while preserving the calculated estimates as variables, allowed us to establish patterns of influence of independent variables on groups of career orientations.

## Results

### Socio-demographic and motivational-professional characteristics of students

Comparison of students by socio-demographic parameters (see Table 2) shows that among respondents in both groups, the majority are girls from middle-income families, with more than half residing in large cities. Among those studying at KFU, there are significantly more students from other regions, which corresponds to the status of the university. This group is dominated by students from large cities or, regional centers. Smolensk State University also has a contingent of students from other regions, primarily those bordering the Smolensk region.

**Table 2:** Socio-demographic and motivational-professional characteristics of interviewees

Variable	Traits	Students KFU (n=83)		Students of SmolSU (n=89)	
		people	%	people	%
Gender:	female	75	90.4	84	94.4
	male	8	9.6	5	5.6
Form of education:	budgetary	70	84.3	57	64.0
	extra-budgetary	13	15.7	32	36.0
Family income:	affluent	10	11.1	13	14.6
	average income	64	77.1	63	70.8
	below average	7	8.4	10	11.2
	low-income	2	4.4	3	3.4
Place of residence:	city — regional center	48	57.8	52	58.4
	town/city — raion center	23	27.7	33	37.1
	rural area	12	14.5	4	4.5
University of enrollment:	own region	45	54.2	69	77.5
	other region	38	45.8	20	22.5
Motive for choosing of education/profession:	conscious	32	38.5	58	65.2
	random	51	61.5	31	34.8
Availability of teaching experience:	no experience	40	48.2	32	36.0
	experienced	43	51.8	57	64.0
Professional plans:	I’m going to work in my specialty	29	34.9	27	30.4
	more likely yes	24	28.9	34	38.2
	depending on circumstances	27	32.5	25	28.1
	no	3	3.6	3	3.4

At SmolSU, more students are enrolled on an extra-budgetary basis, which accounts for the slightly higher percentage of well-offfamilies and the smaller proportion of those living in rural areas. The distribution between the forms of education of students from their own and the other regions at SmolSU is approximately proportional across different forms of education. In contrast, at KFU, students enrolled in paid programs are predominantly from the other regions.

There are a number of differences in motivational-professional characteristics. Among KFU students, almost two thirds of the respondents indicated the main reason for choosing their direction of training was due to accidental circumstances, with the vast majority of these students enrolled on a budgetary basis. In contrast, only 35% of Smolensk State University students reported accidental circumstances as their main reason for choosing their direction of training 94% of the total number of admitted students randomly), while 65% of Smolensk State University students report chosing their field of study consciously. These indicators correspond to the responses regarding the presence of various forms of pedagogical experience before entering the university: 52% of KFU and 64% of SmolSU students reported having such experience. It is also important to note the less significant differences between the universities in terms of students’ plans to work in their profession: 64% of respondents from KFU and 68% from SmolSU intend to work in the field of education. At SmolSU indicators generally correspond to the data on conscious enrollment motivation and the presence of pedagogical experience. The high percentage of KFU students may be explained by the fact that first year students undergo practical training. More than 60% of KFU respondents who entered for random reasons expressed an interest and desire to explore the teaching profession.

To identify patterns when comparing various variables, hierarchical log-linear analysis (see Table 3) was used. It was found that only a few socio-demographic variables are interrelated: in different types of universities, the number of students from other regions significantly differs depending on whether they are admitted to budget-funded or extra-budgetary placements.

**Table 3:** Results of log-linear analysis

Effect	Chi squared test
University*Region	10.448*
Region*Form of education	6.287*
University*Place of residence*Motive	10.156*
Motive*University	11.769*
Motive*Experience*Professional plans	10.100*
Motive*Experience	5.644*
Motive*Professional plans	16.805**
Experience*Professional plans	18.024**

Note:* p<.05; ** p<.001

In contrast, motivational-professional variables showed close connections both between pairs and in aggregate, indicating that conscious admission to a pedagogical program is associated with the presence of pre-university experience in this field and plans to work in the obtained specialty.

Additionally, connections between the type of university and motivation were recorded, confirming significant differences in conscious motivation for admission between students of federal and regional universities.

### Career orientations of students

Comparison of mean scores of Schein’s questionnaire shows values of 5.1 points and higher on all nine scales for students from both universities (see Table 4). This demonstrates the overall variability of career orientations among young people choosing teacher education.

**Table 4:** Descriptive statistics of the results of the Career Anchors questionnaire

Scale	KFU (n=83)		SmolSU (n=89)	
	Average	SD	Average	SD
Professional competence	6.1	1.36	6.2	1.76
Management	6.7	1.91	6.2	1.99
Autonomy	7.3*	1.43	6.7*	1.82
Job stability	7.9	1.55	8.1	1.64
Stability of place of residence	5.7*	2.20	5.1*	1.94
Service	8.1	1.43	8.2	1.50
Challenge	6.1	1.59	5.9	1.92
Lifestyle integration	7.7	1.29	7.7	1.53
Entrepreneurship	6.7**	1.86	5.5**	2.06
Cronbach’s Alpha	0,826		0,823	

Note: * p<.05; ** p<.001

Significant differences were recorded on the scales for *autonomy*, *entrepreneurship*, and *residential stability*, with the mean scores being higher among students from KFU. These differences can be attributed, firstly, to the larger number of non-local students who are forced to adapt to independent living and may face difficulties in doing so, hence the higher scores for *residential stability* and *autonomy*. Secondly, KFU students with high entrance scores and uncertain motivation may exhibit high aspirations, readiness to create new things, and overcome difficulties, which correlates to significantly higher scores for *entrepreneurship*.

No significant differences were found on the other scales. The heist scores (>7) in both groups are observed for career orientations *service*, *job stability*, *lifestyle integration*. This indicates that students receiving pedagogical education, regardless of the university, perceive the teaching profession as significant for societal development (as reflected in the *service* scale) and consider it in demand and supported by social guarantees (as reflected in the *job stability* scale). The high scores on the *lifestyle integration* scale are explained by the clear predominance of young girls (about 90%) who are oriented towards combining gender and professional roles.

Sufficiently high scores on the *autonomy* scale in both groups may be due to the age characteristics of the subjects, This can be considered in the context of general adolescent patterns such as the formation of their own subjective position, or as a characteristic of this generation (*Generation Z*), which prefers work without rigid frameworks allowing for self-actualize and personal enjoyment (Lotkin & Slizhevskaya, 2019).

### The influence of socio-demographic and motivational-professional characteristics on career orientations

It was important for us to determine whether different types of factors affect students’ career orientations, and whether this influence is similar across different types of universities. Therefore, the next step in our analysis was to identify groups of universities from the overall samples for comparing career orientations based on each variable (see *Table 2*). The use of the t-test for independent samples revealed several differences in socio-demographic and especially motivational-professional factors across most of the Schein methodology scales. Variables that showed the influence of factors on career orientation are summarized in *Table 5*.

**Table 5:** T-test for the scales of the Career Anchors methodology based on socio-demographic and motivational-professional variables across different universities

Scale	KFU (n=83)		SmolSU (n=89)	
	Variable	Difference	Variable	Difference
**Professional competence**	Motive (*conscious / random*)^1^	.71164*	Motive (*conscious / random*)	.88409*
			Experience (*yes/no*)	.89079*
	Plans (*going to work / depending on circumstances, no*)	1.20230*	Plans (*going to work / depending on circumstances, no*)	2.17169**
			Plans (*going to work / more likely yes*)	.86035*
	Plans (*going to work / more likely yes*)	.71667*	Plans (*more, likely yes / depending on circumstances, no*)	1.31134*
**Management**	*-*	–	Family income (*low-income / average income*)	–1.26593*
			Experience (yes/no)	1.02007*
			Plans (*going to work / depending on circumstances, no*)	1.25344*
**Autonomy**	Motive (*conscious / random*)	–.63811*	*–*	*–*
**Job stability**	Form of education (*budgetary / extra-budgetary*)	–1.55897*	Place of residence (*regional center / raion center*)	–1.04235*
**Stability of place of residence**	Plans (*going to work / depending on circumstances, no*)	1.65172*	Plans (*more likely yes / depending on circumstances, no*)	1.11765*
**Service**	Plans (*going to work / depending on circumstances, no*)	.76598*	Motive (*conscious / random*)	1.03560*
			Plans (*going to work / depending on circumstances, no*)	1.49180**
			Plans (*more likely yes / depending on circumstances, no*)	1.20378*
**Challenge**	*–*	*–*	Plans (*more likely yes / depending on circumstances, no*)	1.12857*
**Lifestyle integration**	Motive (*conscious / random*)	–.60282*	Motive (*conscious / random*)	.69399*
**Entrepreneurship**	*–*	*–*	Family income (*low-income / average income*)	–1.58071*
			Family income (*low-income / affluent*)	–2.13846*
			Plans (*more likely yes / depending on circumstances, no*)	1.38277*

Note: * p<.05; ** p<.001 ^1^ *In parentheses are discrete values of the factor variables determining differences in mean scores of career orientation scales*

In both groups, no significant differences were found in variables gender and region of admission. Among KFU students, career orientations also do not significantly differ by variables such as family income, place of residence, and teaching experience. Similarly, among SmolGU students, there are no differences by the variable of the form of study.

Among Kazan Federal University students, only one variable from the group of socio-demographic factors, *the form of education*, shows a correlation with the *job stability* scale: students studying on a budgetary basis tend to prefer working in stable organizations. For Smolensk State University students, socio-demographic indicators differentiate the career orientations of *management*, *job stability* and *entrepreneurship*. Students from small localities or those studying on an extra-budget basis are focused on more stable employment. Students from low-income families are less likely to engage in management work or start their own business.

Compared to socio-demographic variables, motivational-professional variables are much more likely to distinguish career orientations, especially those related to teaching. Students from both universities who consciously choose the pedagogical direction of training show higher scores on the *professional competence* scale. Certainty in professional plans positively influences not only this orientation but also *service* and “job stability”.

The following differences across universities were identified. KFU students who randomly entered the pedagogical program have higher scores on *autonomy* and *lifestyle integration*, which may indicate uncertainty in the choice of a future profession for this group. SmolSU students, on the contrary, have higher rates of *integration* among those who consciously chose their field of study. Students at a regional university with previous teaching experience show higher scores on the *professional competence* and *management* scales, indicating the positive impact of early inclusion in practical activities on their understanding of professional development and the importance of management skills and responsibility in the work of a teacher. Also, among students in this group, high scores on the *management*, *challenge* and *entrepreneurship* scales are associated with the desire to work in their chosen specialty. These orientations are integrated into their perception of the teaching profession.

In general, students of the regional higher education institution, who chose the pedagogical sphere for themselves. They also realize the social usefulness and status of their chosen profession and are ready to overcome difficulties and solve complex problems. In turn, the students at federal university, who have not determined their professional future, consider the pedagogical profession only in the context of its external characteristics: social significance and stability.

### Factor analysis

To further compress the data and identify patterns of influence of variables on career orientations, a factor analysis of the scales was carried out. A KMO (Kaiser–Meyer–Olkin criterion) value of 0.797 demonstrates acceptable sampling adequacy. Bartlett’s test of sphericity shows a statistically significant result of 601.444 (p <.001). As a result of the varimax rotation procedure, two factors were identified that explained 58.992% of the variance (see Table 6).

**Table 6:** Results of factor analysis of the “Career Anchors” questionnaire for the entire sample

Factor no.	Dispersion, %	Scales and values	
**1**	30.311	entrepreneurship	.894
		management	.821
		autonomy	.778
		challenge	.604
**2**	28.681	service	.739
		professional competence	.734
		job stability	.656
		lifestyle integration	.600
		stability of place of residence	.587
		challenge	.551

The first factor included orientations related to the changeable and careerist quadrants (Wils et al., 2014), which are linked to with greater levels of activity, initiative, and independence. The second factor included mainly orientations from the social and bureaucratic quadrants. The highlighted factors demonstrate which orientations participants associate with teaching activities and which they consider unrelated to teaching. The inclusion of the *challenge* orientation in both factors can be interpreted as readiness to overcome difficulties and solve complex tasks, which is important for any professional activity, including teaching.

Highlighting computed scores into variables and using the t-test allowed us to identify clear differences between factors, determining the influence of socio-demographic and motivational-professional variables (see *Table 7*).

**Table 7:** Average difference across socio-demographic and motivational-professional variables for factors

Variables	Factor 1	Factor 2
**Socio-demographic:**		
Gender (male / female)	–.14307163	.09771115
Form of education (budgetary / extra-budgetary)	.11106988	–.04672372
Family income (low-income / average income)	–.58620889	.07699762
Family income (low-income / affluent)	–.54031498	.08065645
Place of residence (regional center / raion center)	.31210136	–.27509365
Place of residence (raion center / rural area)	–.63399434*	.12261016
University of enrollment (own region / other region)	–.07843196	–.11504025
University (KFU / SmolSU)	.44227828*	–.12548736
**Motivational-professional:**		
Motive (conscious / random)	–.15649652	.33618986*
Availability of teaching experience (yes/no)	.11916852	.21741136
Plans (going to work / depending on circumstances, no)	.00850186	.79936503**
Plans (more likely yes / depending on circumstances, no)	.07896609	.46462872*

Note: * p<.05; ** p<.001

Based on the data obtained, there is a clear trend where socio-demographic variables show more significant differences in Factor 1 while motivational-professional variables are grouped in Factor 2. The socio-economic status of the family, region, place of residence do not significantly influence attitudes towards the teaching profession and their readiness to work as a teacher. This suggests that contextual factors have a much smaller impact on career choice compared to personality factors.

Regarding socio-demographic characteristics, significant differences are associated with the variable of place of residence, indicating that students from rural areas are less likely to show initiative, and strive for independently organized activities. Significant differences were identified between universities, indicating that more students enter the federal university, which is selective and highly rated, with a focus on independent activity and aspiration for managerial worklit is obvious that among these students, there are more individuals who view the teaching profession as one of several potential career paths, with diverse options for their professional development.

The scales that make up the second factor are related to motivational-professional variables: students at both universities who consciously chose their specialty and plan to pursue a career in it have higher scores on the corresponding career orientations. The most significant differences are observed between those who plan to work in the profession and those who are still uncertain in their career choice. The variable *teaching experience* does not significantly influence these career orientations. It is evident that prior engagement in teaching activities positively impacts the choice of the teaching profession, but its absence is compensated for by the educational process.

The analysis shows that career orientations related to teaching activities naturally interact with the motivational and professional characteristics of students, regardless of the university. However, the overall socio-demographic differences among students shows that more students at KFU are entering the teaching specialty with a focus on independence, leadership roles and a willingness to take initiative.

## Discussion

RQ1. The study found similarities between the groups of students from federal and regional in terms of gender, economic status and place of residence of families, which confirms the high homogeneity of socio-demographic characteristics of students (Shibanova et al., 2023; Zamyatnina, 2021) entering teacher education programs (Quality of admission to Russian universities, 2021). The fact that the federal university enrolls a significant number of students from other regions is explained by its status and quality of education. Additionally, the large number of budget places gives the opportunity to enroll well-performing students from middle- and low-income families living not only in large cities, but also in rural areas. However, it can be assumed that the opportunity to get higher education at the expense of budget places becomes the leading motive to the detriment of an informed professional choice (Minina & Pavlenko, 2023), which is especially noticeable at a federal university. Many students choose the opportunity to study at a high-status university, and the choice of field of study is secondary and is determined by favorable admission conditions. On the other hand, the certainty in the choice of teaching profession among students of a regional university is consistent with the data of the study - students with lower USE scores more often indicate interest in the chosen profession, which is associated with the certainty of their career plans (Shibanova et al., 2023).

RQ2. The study revealed career orientations correlated with the teaching profession among students at both universities: service, workplace stability and lifestyle integration. Service and integration are included in the social quadrant of the career anchors structure, while stability refers to the bureaucratic quadrant (Wils et al., 2014). This indicates that the teaching profession is perceived as collective in nature, combining values of interpersonal relationships with attention to social norms. Similar results were obtained in an earlier study conducted by the authors with over 600 first-year undergraduate students, all future teachers, from 6 Russian universities of different types (Valeeva et al., 2022). High scores on these scales among first-year students at pedagogical universities have been revealed in the studies by Tsaritsentseva (2014), who found high scores in stability and service; and Solovyova & Zausenko (2015), who reported high scores in stability of residence and lifestyle integration. We can assume the existence of a stable *career profile* of a teacher. However, this idea requires further research with the accumulation of data not only on career orientations of students at different stages of education, but also the study of teachers with different lengths of service. It is also advisable to compare career orientations of students and teachers of different specializations in order to clarify whether there are invariant career orientations of pedagogical activity in general and their subject variations.

RQ3. One of the objectives of the research was to identify the factors, both personal and contextual, that influence the career orientations of students who are future teachers. The results show that individual socio-demographic factors influence career orientations, such as management, entrepreneurship, and job stability which are not predominant in the chosen field of activity:.

Personal factors, such as conscious motivation in choosing education and plans for future professional activities, on the contrary, demonstrate an influence not only on the aforementioned orientations career orientations of service and stability, but also on the orientations of professional competence, management and challenge. These factors contribute to professional development and the formation of important qualities for a teacher. However, analysis of the data from the combined sample did not reveal any differences by university. Our findings are consistent with the results of Sheveleva (2019), who found that the career orientation variables concerning *service*, *challenge*, and *professional competence* are the most significant motivational factors. An important aspect in the further study of students’ career orientations is their degree of awareness. It is reasonable to study this in the dynamics from freshmen to graduates as suggested by Tsaritsentseva (2014).

Discussing the findings in the context of the FIT-Choice model (Watt & Richardson, 2007), it is possible to correlate *service* orientation with social utility values, *lifestyle integration* and “job stability” with personal utility values, *professional competence* and possibly *challenge* with intrinsic values. Accordingly, more conscious career motivation to the teaching profession includes not only social and extrinsic motives, but also intrinsic motives.

RQ4. Our study revealed differences in career orientations among students attending universities of different types. The university factor is a stronger differentiating factor than individual socio-demographic characteristics; it explains higher scores on the entrepreneurship, management, and autonomy scales among students at federal universities compared to regional ones. These confirmed differences stem from the fact that many students entering federal universities are clearly motivated by the intention to obtain education at a prestigious institution, and their choice of specific study fields may be driven by external motivation. In contrast, two-thirds of students at regional universities, enter their studies with an established preference for pedagogical fields of study. At the same time, the type of university does not have a significant impact on the career orientations of students motivated towards teaching professions.

Taking into account the influence of the type of university on the career orientations of students destined to be future teachers appears to be productive, as it allows for a comprehensive analysis of various factors, ranging from socio-demographic aspects, which reflect objective parameters and set the social context of the study, to motivational-professional factors, which determin its substantive aspect. Further research of career orientations should include as variables learning profiles, as well as features of the organization of theoretical and practical training in universities of various types.

## Conclusion

In general, according to the results of the study, the career orientations of students from both universities can be considered positive prerequisites for the formation of stable career preferences and conscious plans to work as a teacher. However, federal university students showed a wider variability of career trajectories and uncertainty in professional choice.

These results are further supported by the grouping the career scales into two distinct factors. The first, *managerial-entrepreneurial* level, is influenced by sociodemographic variables, primarily the university. The orientations of the *professional-pedagogical* factor showed a connection with motivational and professional characteristics. Students who consciously choose the pedagogical profession, demonstrate not only beliefs in its high social significance, but also greater readiness for professional development, the overcoming of difficulties, the development of organizational skills, and initiative.

In the context of the anchor model, diagnosing the characteristics of students’ career orientations already at the initial stage of professional training plays an important prognostic function. These characteristics should be taken into account when constructing individual educational trajectories of students, combining theoretical and practical training to stimulate the development of professional values and attitudes, and the formation of interest in the teaching profession. Within the framework of the social-cognitive approach, it has been established that personal and contextual factors have an unequal impact on the professional development of an individual. Also, the university itself, from the standpoint of its status and quality of education, is the most important environmental factor that creates conditions for professional development.

The ability to link the location of vocational education with employment prospects and career retention is essential at the initial stage of vocational training (Jackson & Wilton, 2017). This linkage has a positive impact on subjective career success (Chang et al., 2023).

The results and materials from the study can be useful for researchers examining the problem of personal and professional development of a teacher during their initial professional education. Additionally, education management, engaged in the design and organization of professional and pre-professional training of teachers, as well as teachers, developing programs for the development of professional career of pre-service teachers may also find the materials useful.

## Limitations

The following limitations should be kept in mind when interpreting the study data. First, although the HEIs described are typical for their categories, using one HEI for each category may include additional unique characteristics not accounted for in the study but influencing the results. For example, the national factor in KFU or the proximity of the capital region for SmolSU. Using data from several universities for each category would reduce this influence. Secondly, the sample of respondents is limited, which may affect the reliability of the results. Thirdly, the compared groups of students have different specializations, which can also influence the differences in career orientations.
